# *Phoenix dactylifera* seed-derived biochar as a sustainable and environmentally feed supplement in camel: impacts gas production, methane emissions, nutrient degradability and fermentation parameters, performance predictions

**DOI:** 10.3389/fvets.2025.1632447

**Published:** 2025-08-18

**Authors:** Hesham S. Ghazzawy, Nashi K. Alqahtani, Abdullah Sheikh, Mohamed Shawky El Sayed, Roshmon Thomas Mathew, Hassan M. Ali-Dinar, Ehab El-Haroun, Mohamed M. A. Abd-Elkarim, Sameh A. Abdelnour, Ali S. A. Saleem

**Affiliations:** ^1^Date Palm Research Center of Excellence, King Faisal University, Al-Ahsa, Saudi Arabia; ^2^Department of Food and Nutrition Sciences, College of Agricultural and Food Sciences, King Faisal University, Al-Ahsa, Saudi Arabia; ^3^Fish Resources Research Center, King Faisal University, Al-Ahsa, Saudi Arabia; ^4^Camel Research Center, King Faisal University, Al-Ahsa, Saudi Arabia; ^5^Avian Research Center, King Faisal University, Al-Ahsa, Saudi Arabia; ^6^Department of Integrative Agriculture, College of Agriculture and Veterinary Medicine United Arab Emirates University, Al Ain, United Arab Emirates; ^7^Animal Production Department, Faculty of Agriculture, Zagazig University, Zagazig, Egypt; ^8^Animal Production Department, Faculty of Agriculture, Sohag University, Sohag, Egypt

**Keywords:** *Phoenix dactylifera* seed-derived biochar, gas production, methane emissions, degradability, fermentation parameters, predicted camel’s performance

## Abstract

**Introduction:**

Climate change poses a significant environmental challenge to all living organisms. Camels exhibit notable resilience to these changes. Concurrently, the date palm (*Phoenix dactylifera*), a widely cultivated plant in tropical and subtropical regions, generates substantial seed waste. Valorizing *Phoenix dactylifera* seed-derived biochar (PSB) to enhance feed supplements and mitigate environmental impacts presents a potentially sustainable and eco-friendly solution. This study investigated the potential of date palm seed-derived biochar as a sustainable feed additive for dromedary camels to reduce methane (CH₄) emissions and improve gas production, nutrient degradability, fermentation parameters, and performance predictions using *in vitro* models.

**Methods:**

The PSB was synthesized and stored at 4°C until use. Ruminal fluids were collected from growing camels (24-36 months old) at the nutrition laboratory and subsequently incubated at 37°C. The basal diet was supplemented with PSB at 0, 1, 2, and 4%, and the resulting data were analyzed using polynomial analysis. Gas production, methane emissions, nutrient degradability, fermentation parameters, and performance predictions were assessed.

**Results:**

At 6, 12, and 36 hours of incubation, all levels of PSB biochar supplementation resulted in a significant linear increase in gas production (*p* < 0.05). The inclusion of PSB significantly reduced CH₄ emissions in a quadratic manner (*p* < 0.001). The lowest reduction in CH₄ production was observed at the 1% and 2% PSB inclusion levels, with a greater reduction at the 4% level (quadratic effect; *p* < 0.001). A significant quadratic increase in TVFA production was observed with increasing PSB inclusion levels during the *in vitro* fermentation of camel diets (quadratic effect; *p* < 0.01). Furthermore, pH values significantly decreased with biochar supplementation, exhibiting a linear trend with the lowest values at the 4% level, followed by 2% and 1% (linear effect; *p* < 0.01). Short-chain fatty acid (SCFA) production was improved by the addition of PSB compared to the control diet in camels (quadratic effect; *p* < 0.01). The inclusion of 1% or 2% PSB quadratically improved organic matter digestibility (%), metabolizable energy (DM), and net energy for lactation (NEL) in camels. Microbial crude protein (MCP) and purine derivatives (PD) were not significantly affected by PSB supplementation (*p* > 0.05).

**Conclusion:**

In summary, the addition of PSB enhanced gas production, nutrient degradability, fermentation parameters, and performance predictions, while concurrently mitigating methane emissions *in vitro*. This study underscores the potential of utilizing PSB as a valuable feed supplement and a sustainable feed additive for dromedary camels in extensive production systems.

## 1 Introduction

Developing cost-effective strategies to convert agricultural residues into valuable products is a global approach that directly addresses waste accumulation, a key contributor to environmental pollution. This aligns with the United Nations Sustainable Development Goals and offers considerable advantages across various sectors, particularly the livestock industry (1, 2). The date palm (*Phoenix dactylifera* L.), belonging to the Arecaceae family, is among the oldest cultivated fruit trees globally, a history in the Middle East and North Africa spanning over 5,000 years (1). Originating in the Persian Gulf region (3), these long-lived trees can exceed a century in lifespan. Major global date production is concentrated in countries such as Egypt, Saudi Arabia, Iraq, Algeria, and Iran (4). Consequently, date palm cultivation generates substantial residual biomass, particularly abundant in the Arabian region (1). This residual biomass is frequently disposed of through open-field burning, a practice that significantly contributes to environmental pollution in date-producing countries. Although some nations have incorporated this biomass into livestock feed, there remains a critical need to develop more sustainable and widely adopted applications for these currently underutilized resources (5, 6).

Given the escalating challenges of global climate change and rising temperatures, exacerbated by growing concerns regarding water scarcity, the date palm has emerged as a strategically significant crop. This is primarily due to its exceptional resilience to adverse climatic conditions and minimal water requirements (6). Furthermore, beyond its nutritious fruit, the date palm provides substantial environmental and economic advantages.

Global date palm cultivation, comprising over 120 million trees, produces substantial quantities of dates and significant secondary biomass, including midribs, fronds, stems, leaves, and coir (4). Over 84 million of these trees are concentrated in Egypt, Saudi Arabia, Algeria, Iraq, Iran, Morocco, and Tunisia (4). These plantations, which occupy approximately 3% of the world’s cultivated land, generate an estimated 12 million metric tons of biomass waste annually (7). Despite this considerable volume, date palm seed residues remain largely underexploited, primarily due to a lack of cost-efficient processing methods (5, 6). More effective utilization of this biomass could not only enhance its economic value but also promote environmental sustainability by mitigating ecological burdens (5).

Several studies have investigated the effects of date palm seeds supplementation in the diets of both terrestrial (8–10), and aquatic (11) animals. The findings suggest potential benefits for animal health and productivity, while also addressing environmental concerns associated with date palm seed waste accumulation (4, 10). However, livestock systems are a significant contributor to global methane emissions, accounting for approximately 18% of the total and thereby exacerbating climate change. To mitigate this impact, various strategies have been introduced, including the incorporation of biochar into animal feed (12, 13).

Biochar, an economical soil enhancer with widespread agricultural applications, is typically synthesized via the thermochemical conversion of agricultural residuals byproducts (14). Biochar’s effectiveness is largely determined by its characteristics, including its surface area, porosity, and the functional groups present on its surface (15). These properties are significantly influenced by the pyrolysis conditions and the original biomass feedstock. A recent area of investigation involves using biochar made from date palm seeds that has been magnetized with Fe₃O₄. This modified biochar shows promise for efficiently removing copper ions (Cu^2+^) from contaminated water solutions (16). Biochar derived from date palm seeds are rich in minerals, carbon, and various fibers (17). Lignin, a prominent fiber in date palm seeds, makes up 21.2 to 24.06% of its composition (18). Research indicates that probiotic-inoculated biochar (at an inclusion rate of 50 g/kg dry matter) used as a dietary supplement for livestock can lead to several improvements. These include enhanced dry matter digestibility, increased microbial protein synthesis, and a higher milk fat concentration, all while maintaining total milk yield (19). Similarly, the inclusion of *Phoenix dactylifera* seed-derived biochar (PSB) in sheep diets has been linked to reduced gas emissions and improved growth performance (13).

Research indicates that biochar derived from waste materials can reduce methane production when added to cattle feed. For instance, A study by Leng et al. (20) reported a 22% reduction in methane production. Furthermore, Winders et al. (21) observed that supplementing cattle diets with 0.8% biochar led to a 9.5 and 18.4% reduction in enteric methane generation (g kg dry matter intake) during the growth and finishing stages, respectively. Similarly, a 0.5% biochar addition to an *in vitro* rumen experiment resulted in a 25% reduction in methane production (22). Adding date palm seed to sheep diets (up to 20%) improved the digestibility, milk yield, and composition in sheep (23) and other ruminant (24). Supplementing ruminant diets with biochar can beneficially alter rumen fermentation, leading to increased propionic acid and decreased methane emissions (25). Nevertheless, other studies suggest that non-inoculated biochar may not significantly impact milk yield, physiological indicators, or methane emissions in dairy cows (12). Given these inconsistent findings, the current *in vitro* experiment aims to assess the impact of a novel *Phoenix dactylifera* seed-derived biochar (PSB) on gas production, methane emission, degradability, fermentation dynamics, and predicted camel performance.

## 2 Materials and methods

### 2.1 Ethical statement

All experimental procedures and animal handling were reviewed and approved by the Animal Use in Research Committee (IACUC) at Zagazig University, Egypt, under approval number ZU-IACUC/2/F/25/2023. Throughout this experiment, all efforts were made to minimize animal suffering in accordance with the ARRIVE guidelines. The present study was carried out in the Laboratory of Animal Nutrition, Animal Production Department, Faculty of Agriculture, Zagazig University, Zagazig, Egypt.

### 2.2 Biochar synthesis

**Figure 1:**
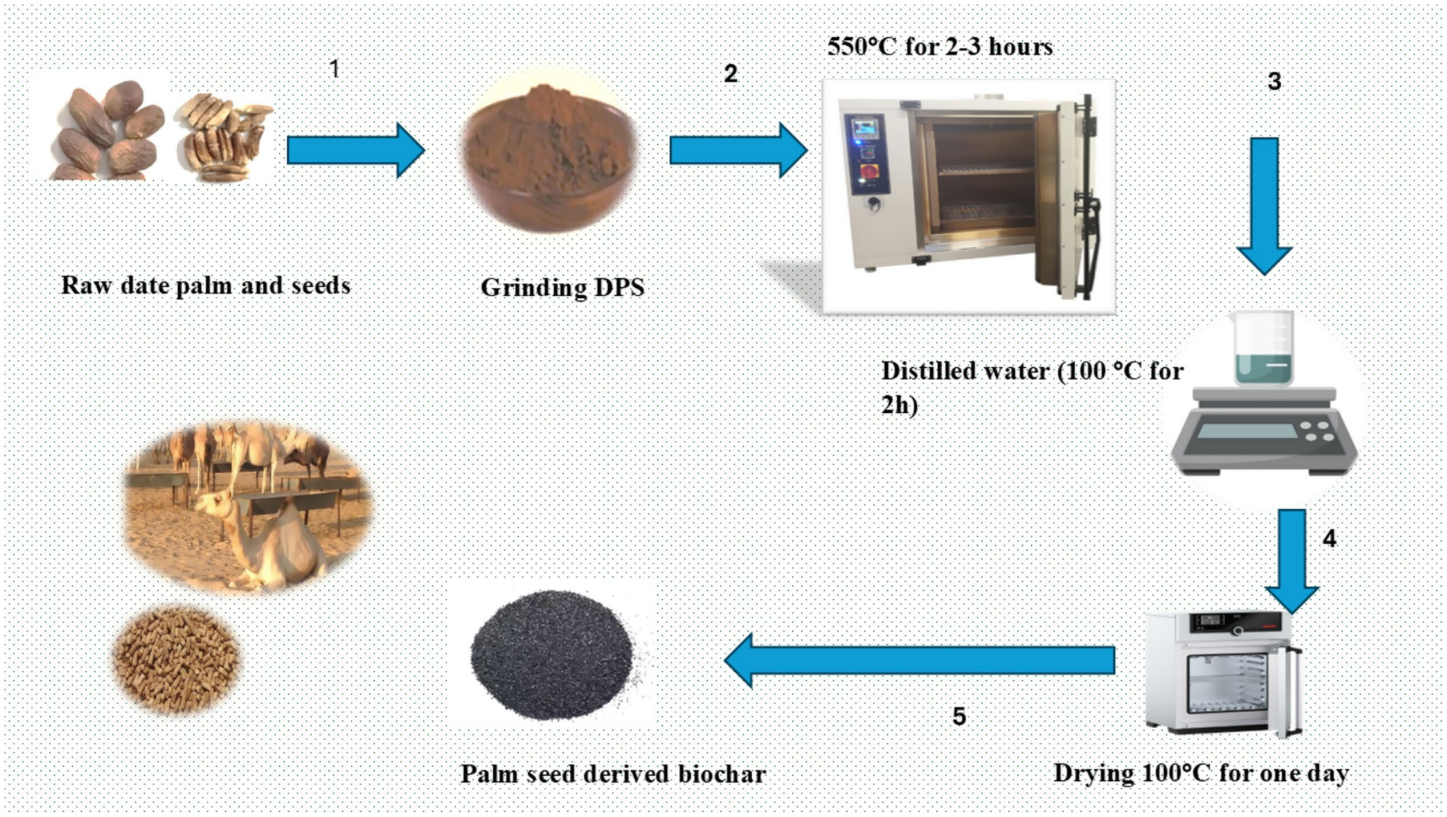
The steps of synthesized date palm seed-derived biochar.

Date palm seeds, sourced from Linah Farm, Monufia Governorate, Egypt,^1^ were chopped and used as feedstock for biochar production. Pyrolysis was performed at 550°C for 2–3 h under oxygen-depleted conditions to produce the biochar (Figure 1). After pyrolysis, the resulting biochar was ground and sieved to obtain a particle size range of 1–2 mm. The pyrolysis process involved two distinct steps, each employing a controlled heating rate of 10°C per minute (16). In the first step, the temperature gradually increased to 300°C and maintained for 1 h. Following this, the system was allowed to cool to room temperature over a 12-h period, facilitating a gradual transition to the subsequent phase. The second step involved raising the temperature to 600°C, which was sustained for 1 h to further enhance the biochar’s characteristics. After this final holding period, the system was again cooled down to room temperature. The two-step pyrolysis process was selected over a single-step approach due to its enhanced control and efficiency in converting biomass to biochar, resulting in the formation of larger and more uniform pores (16). The resulting biochar was then ground and sieved to a particle size of 2 mm for uniformity. The processed biochar was stored in a dry environment until its use in this experiment (26). The yield was stored in a dry environment until used in this experiment.

### 2.3 Diet, treatment and chemical analysis

Four experimental diets were formulated, supplemented with *Phoenix dactylifera* seed-derived biochar (PSB) at levels of 0% (control), 1% (PSB1), 2% (PSB2), and 4% (PSB4). The basal substrate for the *in vitro* fermentation consisted of 30% berseem hay (*Trifolium alexandrinum*) and 70% concentrate mixture. The concentrate and berseem hay were finely ground (1 mm) and mixed at a ratio of 30:70, respectively. This dried substrate was used for both chemical analysis and *in vitro* gas production studies; the chemical composition of the substrate is detailed in Table 1. Biochar was added to the diet at 1, 2, and 4%, replacing an equivalent percentage of berseem hay. The substrate was analyzed for dry matter (DM), ash, organic matter (OM), ether extract (EE), and crude protein (CP) according to the methods of the Association of Official Analytical Chemists (27). Neutral detergent fiber (NDF) content was determined using the method described by Van Soest et al. (28). In brief, feed samples are ground to 0.1 mm and boiled in a neutral detergent solution containing sodium lauryl sulfate, EDTA, sodium borate, disodium hydrogen phosphate, 2-ethoxyethanol, heat-stable amylase, and sodium sulfite. This process solubilizes cell contents like sugars, starches, proteins, and lipids. The remaining insoluble residue, primarily hemicellulose, cellulose, and lignin, represents the NDF. This residue is then filtered, washed with hot water and acetone, dried, and weighed. The NDF content is calculated from the initial sample weight and the final residue weight.

**Table 1:** Formulation and chemical composition of the concentrate mixture, berseem hay, and basal diet.

Ingredients	Kg/Ton
Yellow corn	385
Soybean meal	154
Wheat barn	126
Common salt	10.5
Limestone	17.5
Sodium bicarbonate	3.5
Mineral and vitamin mixture^a^	3.5
Berseem hay	300

Chemical composition (%, on DM basis)	
Nutrient	basal diet^b^
Organic matter	94.81
Crude protein	15.96
Ether extract	2.98
Neutral detergent fiber	29.21
Acid detergent fiber	16.66
Ash	4.84
Crude fiber	12.70
Nitrogen free extract	63.18

a Minerals and vitamins mixture contained: Copper 30,000 mg, Iodine 800 mg, Selenium 300 mg, Iron 10,000 mg, MgO 80,000 mg, Zinc 100,000 mg, Cobalt 400 mg, Vit. A 10000000 IU, Vit. D_3_ 2,500,000 IU, Vit. E 35000 IU, and CaCO_3_ to 3 Kg.

b The basal diet was a total mixed ration containing 50% Berseem hay (Trifolium alexandrinum) and 50% concentrate mixture. Biochar was added to the diet at 1, 2, and 4%, replacing an equivalent percentage of berseem hay.

### 2.4 *In vitro* incubations

Ruminal fluid was collected from a slaughtered camel at a slaughterhouse located in Zagazig, Sharkia Governorate, Egypt according to the method of Lutakome et al. (29). The rumen fluid was rapidly transported to the laboratory in a pre-warmed (39°C) insulated flask and maintained under anaerobic conditions until use. Upon arrival, the rumen fluid was filtered through four layers of cheesecloth, then incubated in a water bath at 39°C and saturated with CO₂ until inoculation.

The buffered incubation medium (MB9) consisted of NaCl (2.8 g/L), CaCl₂ (0.1 g/L), MgSO₄·7H₂O (0.1 g/L), Na₂HPO₄ (6 g/L), and KH₂PO₄·H₂O (2 g/L). The pH of the MB9 medium was adjusted to 6.8, and anaerobic conditions were maintained by flushing with CO₂ for 30 min (30). The MB9 medium was mixed with the filtered rumen fluid at a 2:1 ratio (v/v). For incubation, glass tubes were used, each loaded with 200 mg of the experimental diet amended with date palm seed biochar (PSB) at various concentrations.

Each tube was injected with 30 mL of the mixed ruminal fluid, immediately sealed with a gas-release rubber stopper connected to a three-way valve and a calibrated plastic syringe for gas volume measurement. Gas production was recorded at 3, 6, 12, 24, 36, and 48 h of incubation. Blank tubes (without substrate) were included to correct for gas production from the inoculum. Each experimental run included four blank bottles and six replicate bottles for each treatment.

At the end of the incubation period, after the final gas volume was recorded, methane emission was estimated by NaOH (10 M) absorption according to Fievez et al. (31). Methane intensity was then calculated and expressed as mL CH₄/g TDDM, mL CH₄/g TDOM, and as a percentage of total gas produced.

### 2.5 Estimation of pH, ammonia-N, volatile fatty acids concentration, partitioning factor, and true nutrient degradation

At the end of the *in vitro* incubation, ruminal pH was measured immediately using a digital pH meter (model 6,010 N, Jenco Instruments Inc., San Diego, CA, USA). Following 48 h of incubation, 30 mL of neutral detergent solution was added to the contents of three replicate tubes per treatment, and the tubes were placed at 105°C for 3 h to determine truly degraded dry matter (TDDM). The residual dry matter weight was then estimated after filtering each sample through pre-weighed Gooch crucibles and drying at 105°C for 3 h (32). This value was subsequently used to estimate crude fiber degradability (CFD) according to (27). The contents of another three replicate tubes per treatment were used to determine the concentrations of ammonia-nitrogen (NH₃-N) and total volatile fatty acids (TVFA). TVFA concentration was determined using the steam distillation method as described by Warner (33). Briefly, to prepare for VFA concentration analysis by steam distillation, 10 mL of rumen fluid was combined with 2 mL of 25% (wt./vol) metaphosphoric acid and then frozen at −20°C to preserve the sample until analysis.

Ruminal NH₃-N concentration was measured using the method proposed by Conway (34). The partitioning factor (PF) was calculated as the ratio of organic matter (mg) degradability to gas production volume (mL at 24 h) (32).

### 2.6 Estimation of nutrients digestibility calculations

The equation of Menke and Steingass (35) was used to calculate The net energy of lactation (NEL, MJ/kg DM) and metabolizable energy (ME, MJ/kg DM).


$$\begin{array}{c} \mathrm{ME}\;(\mathrm{MJ}/\mathrm{kg}\;\mathrm{DM})=(0.157\times \mathrm{GP})+(0.0084\times \mathrm{CP})\\ +(0.022\times \mathrm{EE})–(0.0081\times \mathrm{CA})+1.06. \end{array}$$

$$\begin{array}{c} \mathrm{NEL}\;(\mathrm{MJ}/\mathrm{kg}\;\mathrm{DM})=(0.115\times \mathrm{GP})+(0.0054\times \mathrm{CP})\\ +(0.014\times \mathrm{EE})–(0.0054\times \mathrm{CA})-0.36. \end{array}$$

Where,

GP = net gas production (mL/0.2 g DM) at 24 h of incubation; EE = ether extract; CP = crude protein; CA = crude ash.

Short-chain fatty acid concentrations (SCFA) were calculated according to Getachew et al. (36) as:

SCFA(mmoL/200mgDM)=(0.0222×GP)−0.00425.

Where GP is the 24-h net gas production (ml/200 mg DM).

Microbial CP biomass production was estimated, according to Blümmel et al. (32) as follows:


MCP(mg/gDM)=mgDMD−(mlgas×2.2mg/mL).

Where: 2.2 mg/mL is a stoichiometric factor that expresses mg of C, H, and O required to produce SCFA gas associated with production of 1 mL of gas.

Menke et al. (37) equation was used to calculate the *in vitro* organic matter digestibility (OMD %) as OMD (%) = 14.88 + (0.889 × GP) + (0.45 × CP) + (0.0651 × XA),

Where XA = Ash (%).

### 2.7 Data analysis

Shapiro–Wilk and Levene’s tests were used to confirm data normality and homogeneity of variance. Statistical analyses were conducted with SPSS 25.0 (SPSS Inc., Chicago, IL, USA) using a mixed-effects model (PROC MIXED). To assess the dose–response relationships (linear and quadratic) of biochar (0, 1, 2, and 4% g/kg diet) on each dependent variable, orthogonal contrasts were applied. Statistical significance was set at *p* < 0.05, and Duncan’s multiple range test was used for post-hoc comparisons.

## 3 Results

### 3.1 Effects of biochar derived from date palm seeds on gas production

At the 3-h time point, the inclusion of *phoenix dactylifera* seed-derived biochar (PSB) in the *in vitro* fermentation of camel diets resulted in a significant linear increase in gas production (*p* < 0.001, Table 2). At 6, 12, and 36 h, all levels of PSB biochar supplementation led to a significant linear increase in gas production (*p* < 0.05). At 24 h, while the 1 and 2% PSB biochar inclusion levels exhibited higher gas production with a quadratic effect, the 4% level did not differ significantly from the other groups (*p* > 0.05). After 48 h, PSB biochar supplementation significantly increased gas production, particularly at the 2 and 4% inclusion levels, whereas the 1% level showed similar gas production to the control group (*p* > 0.05).

**Table 2:** Dose–response effects of date palm seed biochar on *in vitro* gas production in camel rumen fluid.

Item	Biochar supplementation				SEM	*p*-value		
	Control	Biochar 1%	Biochar 2%	Biochar 4%		*ANOVA*	*Lin.*	*Quad.*
Gas production, mL/g DM								
3 h	21.67^d^	26.25^c^	36.67^a^	35.00^ab^	1.924	0.007	0.001	0.320
6 h	47.92^b^	57.92^a^	60.42^a^	57.92^a^	1.728	0.039	0.026	0.052
12 h	76.67^b^	85.83^a^	85.42^a^	85.00^a^	1.391	0.045	0.037	0.065
24 h	110.00^b^	122.50^a^	124.58^a^	117.92^ab^	1.867	0.017	0.079	0.006
36 h	136.25^b^	148.33^a^	152.08^a^	152.08^a^	2.124	0.014	0.004	0.101
48 h	149.1^7b^	160.42^ab^	166.67^a^	162.92^a^	2.262	0.027	0.013	0.067

Polynomial contrasts to determine the p-values for the ANOVA (ANOVA) and the linear (Lin.) and quadratic (Quad.) effects of different biochar addition levels (0, 1, 2, and 4%) were used in this study. Means within the same row with different letters (^a, b^) are significantly different (*p* < 0.05). The pooled standard error of means (SEM).

### 3.2 Effects of biochar derived from date palm seeds on methane emissions

The inclusion of PSB significantly reduced methane (CH₄) emissions in a quadratic manner (*p* < 0.001, Table 3). The lowest reduction in CH₄ production (mL/g DMD or mL/g OMD) in response to date palm seed-derived biochar was observed at the 1 and 2% inclusion levels, followed by a greater reduction at the 4% level (quadratic effect; *p* < 0.001). No statistically significant difference was found between the 2 and 4% inclusion levels for CH₄ production expressed per gram of OMD or as a percentage of total gas produced (*p* > 0.05). Furthermore, the incorporation of PSB-derived biochar into camel diets reduced the CH₄ proportion of total gas production (GP) in a quadratic manner (*p* < 0.001).

**Table 3:** Dose–response effects of date palm seed biochar on *in vitro* methane emission in camel rumen fluid.

Item	Biochar supplementation				SEM	*p* values		
	Control	Biochar 1%	Biochar 2%	Biochar 4%		*ANOVA*	*Lin.*	*Quad.*
CH_4_ mL/ 1 g	53.33^a^	32.08^c^	36.00b^c^	40.83^b^	1.971	<0.0001	0.003	<0.0001
CH_4_ mL / g DMD	84.89^a^	50.46^c^	56.77^bc^	64.91^b^	3.172	<0.0001	0.003	<0.0001
CH_4_ mL / g OMD	127.12^a^	72.56^c^	80.91^bc^	94.12^b^	4.866	<0.0001	<0.0001	<0.0001
CH_4,_ % of GP	35.74^a^	19.98^c^	21.58^bc^	25.08^b^	1.399	<0.0001	<0.0001	<0.0001

Polynomial contrasts to determine the p-values for the ANOVA (ANOVA) and the linear (Lin.) and quadratic (Quad.) effects of different biochar addition levels (0, 1, 2, and 4%) were used in this study. Means within the same row with different letters (^a, b, c^) are significantly different (*p* < 0.05). The pooled standard error of means (SEM). Total dry matter degradability (TDDM).

### 3.3 Effects of biochar derived from date palm seeds on degradability and fermentation parameters

The effects of date palm seed-derived biochar inclusion on nutrient degradability are presented in Table 4. The incorporation of various levels of PSB-derived biochar did not significantly affect dry matter degradability (DMD; *p* = 0.522, Table 4) or crude fiber degradability (CFD; *p* = 0.07) in the *in vitro* fermentation of camel diets. In contrast, ammonia-nitrogen (NH₃-N) concentrations were influenced by biochar supplementation, with the highest values observed at the 4% inclusion level, while the lowest values were noted at the 2% level. No significant differences in NH₃-N concentrations were detected between the 1% biochar level and the control diet; however, the 4% level exhibited significantly higher NH₃-N concentrations (*p* < 0.05). Notably, a significant quadratic increase in total volatile fatty acid (TVFA) production was observed in response to increasing inclusion levels of PSB-derived biochar in the *in vitro* fermentation of camel diets (quadratic effect; *p* < 0.01). Furthermore, the pH values significantly decreased with biochar supplementation, showing a linear trend with the lowest values at the 4% level, followed by 2 and 1% (linear effect; *p* < 0.01).

**Table 4:** Dose–response effects of date palm seed biochar on *in vitro* degradability and fermentation parameters in camel rumen fluid.

Item	Biochar supplementation				SEM	P value		
	Control	Biochar 1%	Biochar 2%	Biochar 4%		*ANOVA*	*Lin.*	*Quad.*
Degradability								
DMD	62.83	63.58	63.41	62.91	0.410	0.92	0.99	0.522
CFD	21.30	24.86	24.23	28.69	1.265	0.24	0.07	0.851
Fermentation parameter								
Ammonia (mg/100 mL)	12.29^b^	12.39^b^	10.08^c^	13.04^a^	0.483	0.013	0.99	0.117
TVFA (mL/L)	174.33^b^	206.00^a^	205.33^a^	201.67^a^	4.422	<0.001	<0.001	0.005
pH	6.31^a^	6.06^ab^	6.01^ab^	5.90^b^	0.055	0.055	0.010	0.486

Polynomial contrasts to determine the p-values for the ANOVA (ANOVA) and the linear (Lin.) and quadratic (Quad.) effects of different biochar addition levels (0, 1, 2, and 4%) were used in this study. Means within the same row with different letters (^a, b^) are significantly different (*p* < 0.05). The pooled standard error of means (SEM). Total volatile fatty acids (TVFA), dry matter degradability (DMD), and organic matter degradability (OMD).

### 3.4 Effects on predictive value

Short-chain fatty acid (SCFA) production was improved by the addition of PSB-derived biochar compared to the control diet in camels (quadratic effect; *p* < 0.01) (see Table 5). The inclusion of 1% or 2% PSB-derived biochar quadratically improved organic matter digestibility (OMD, %), metabolizable energy (ME, MJ/kg DM), and net energy for lactation (NEL, MJ/kg DM) in camels. In contrast, the high inclusion level (4%) did not result in significant differences in OMD (%), ME (MJ/kg DM), and NEL (MJ/kg DM) in camels compared to the other treatments (*p* > 0.05). Microbial crude protein (MCP, mg/g DM) and purine derivatives (PF, mg TDOM/mL gas) were not significantly affected by the addition of PSB-derived biochar to camel diets (*p* > 0.05).

**Table 5:** Dose–response effects of date palm seed biochar on *in vitro* predictive value in camel rumen fluid.

Item	Biochar supplementation				SEM	P values		
	Control	Biochar 1%	Biochar 2%	Biochar 4%		*ANOVA*	*Lin.*	*Quad.*
SCFA (mmol)	0.48^b^	0.54^a^	0.55^a^	0.52^a^	0.008	0.016	0.064	0.006
ME (MJ/Kg DM)	4.67^b^	5.07^a^	5.13^a^	4.92^ab^	0.059	0.017	0.081	0.006
NE_L_ (MJ/Kg DM)	2.27^b^	2.56^a^	2.61^a^	2.45^ab^	0.043	0.017	0.081	0.006
OMD (%)	41.94^b^	44.16^a^	44.53^a^	43.34^ab^	0.332	0.018	0.080	0.006
MCP (mg/g DM)	581.36	583.04	579.81	576.31	4.672	0.97	0.71	0.816
PF (mg/TDOM/ mL gas)	1.95	1.82	1.80	1.82	0.027	0.17	0.09	0.142

Polynomial contrasts to determine the p-values for the ANOVA (ANOVA) and the linear (Lin.) and quadratic (Quad.) effects of different biochar addition levels (0, 1, 2, and 4%) were used in this study. Means within the same row with different letters (a, b) are significantly different (*p* < 0.05). The pooled standard error of means (SEM). Short-chain fatty acids (SCFA), metabolizable energy (ME), net energy lactation (NEL), microbial crude protein production (MCP), organic matter degradability (OMD), partitioning factor (PF) at 72 h of incubation.

## 4 Discussion

Climate change poses a significant environmental threat to all living things. In this context, camels demonstrate a degree of resilience. Interestingly, the extensive cultivation of date palms (*Phoenix dactylifera*) across tropical and subtropical regions produces a substantial amount of seed waste. A promising sustainable and eco-friendly strategy involves transforming this waste into biochar from PSB (38). This PSB can then be used to enhance animal feed supplements, simultaneously reducing ecological impact. For some time, various strategies have been explored to mitigate the effects of environmental stressors on livestock, including the use of phytochemicals, organic acids, and probiotics. Coinciding with these efforts, date palm cultivation has continued to generate substantial byproducts, particularly date palm seeds. These seeds have already shown success when incorporated into livestock feed, maintaining animal performance and productivity. Transforming the date palm seed waste into biochar is a game-changer (38). It tackles a major waste problem head-on and offers a powerful one-two punch: it helps make livestock, like camels, more resilient by boosting their feed, and it contributes to a much more sustainable agricultural system overall.

This *in vitro* study explored the use of date palm seed-derived biochar (PSB) as a feed additive for dromedary camels. Researchers found that PSB supplementation increased gas production and improved nutrient digestion and fermentation parameters. Notably, PSB quadratically reduced methane emissions, with the highest reduction at a 4% inclusion level. Supplementation with PSB led to increased TVFA and SCFA production, alongside a reduction in ruminal pH. Optimal enhancements in feed digestibility and energy values were evident at 1 and 2% PSB inclusion levels. While MCP and PD values were not significantly altered, these findings suggest that PSB holds potential as a sustainable feed additive for camels. Its application could contribute to improved animal productivity and a reduction in their environmental footprint, primarily through attenuated methane emissions.

Biochar consists of many minerals, such as potassium (K), phosphorus (P), sulfur (S), and calcium (Ca) (39). With the global population continuously expanding and anticipated to reach around 10 billion by 2,100, ensuring future food and water security remains a formidable challenge. Overcoming this challenge requires the promotion of sustainable agriculture and the intensification and expansion of both livestock and crop production to meet the growing demand for food (40, 41).

Dromedary camels are well-adapted livestock for harsh environments, capable of producing milk and meat and reproducing efficiently. Moreover, camels are considered non-conventional ruminants with distinctive features in their digestive physiology, particularly in the composition and activity of the rumen microbiome, making them of ecological and economic importance in arid and semi-arid regions (42). Addition, camels are playing an important role in utilizing low-quality forages efficiently due to their unique rumen microbial composition (43). The present study’s findings demonstrate that the inclusion of biochar derived from PSB in camel diets led to substantial improvements in gas production dynamics, a pronounced decrease in methane emissions, and advancements in fermentation parameters and nutrient utilization efficiency. Our findings align with previous research suggesting that biochar plays a dual role in reducing methane emissions. Firstly, biochar provides a habitat that promotes the growth of methanotrophs (15, 20). These crucial microbes oxidize methane within the gut, directly leading to a reduction in released methane. Secondly, biochar’s inherent ability to adsorb and absorb gases (15) is another significant factor in lowering enteric methane production.

The data showed that the addition of PSB to growing camel diets significantly increased gas production at most points of time. A linear increase (*p* < 0.05) was observed at 3, 6, 12, and 36 h of incubation, while at 24 h, a quadratic response (*p* > 0.05) showed at 1 and 2% levels with no significant difference at 4%. At 48 h, gas production significantly (*p* > 0.05) increased at 2 and 4% levels, but 1% showed no difference from the control. Date palm seed (PSB) biochar supplementation significantly enhanced gas production in growing camel diets over time, especially at 2 and 4% inclusion levels, this delayed increase in gas production can be attributed to the porous structure and high adsorption capacity of biochar, which contribute to the stabilization of microbial communities and the enhancement of enzymatic digestion (44). Similar patterns have been previously reported in cattle and sheep fed various types of biochar (21, 45).

Our study found that adding palm seed-derived biochar to the diets of growing camels significantly reduces methane emissions. Specifically, methane emissions were reduced by 39.85, 32.50, and 23.44% when biochar was supplemented at 1, 2, and 3% levels, respectively. The most substantial reduction occurred with just 1% biochar inclusion. This aligns with previous research suggesting that biochar works by altering microbial hydrogen pathways, thereby redirecting electrons away from methanogenesis (44, 46). Addition, camel rumen fluid harbors a uniquely structured microbial population, characterized by a higher abundance of highly efficient methanogens; the inhibitory effect of biochar on these communities may be more pronounced in camels than in cattle or sheep (47). These findings are consistent with previous studies involving biochar supplementation in sheep diets, which reported methane reductions between 65.58 and 78.39% (13). Also, incorporating biochar into dairy manure has methane reductions close to 58% (48), while other investigator fed sheep inoculated biochar registered a 9% reduction in methane emissions than controls (41).

Meta-analyses and controlled studies show that biochar can reduce methane emissions by an average of 21% in ruminants, but results vary widely depending on the type, dose, and delivery method of biochar used (49–51). Some controlled pen trials in cattle have shown modest reductions in methane emissions (8.8–12.9%) without negative effects on feed intake or fermentation, but these effects were not observed in grazing conditions (50).

Beyond simple pyrolysis, treating biochar with mineral salts or weak acids significantly boosts its properties. This process, called chemical activation, increases the biochar’s pore size and surface area. It also adds various functional groups, like organic acids and phosphate groups, which in turn enhance the biochar’s adsorption capabilities and chemical characteristics (15, 52). Additionally, acidic biochars have been found to enhance interspecies hydrogen transfer among microbial populations. This is especially advantageous in environments like the rumen, as it can significantly boost microbial activity and fermentation (53, 54). The facts support biochar’s argument of lowering methane emissions; however, confirming this with other studies remains inconsistent. Some author’s reported that no conclusions from the use of pine derived biochar and its effects on methane emissions and milk production of cattle (8, 55, 56). In contrast, another study showed a 40% reduction of methane emissions with the addition of 0.6% of biochar added to the diet of cattle (20). A study determined that date seeds are successful in producing porous biochar due to their properties, such as low ash (1.14%), high volatile matter (65%), and high bulk density (0.5 g/mL) (17). It’s a high-value material that can be used as a soil amendment and for energy generation, which helps mitigate climate change (57).

Although specific studies on date palm seed biochar are sparse, one study noted that it did improve growth, nutrient digestibility, and health in sheep, which was observed (13). In contrast, other studies highlight the need for more extensive *in vivo* studies. For instance, Winders et al. did not observe a reduction in methane yield for steers on finishing diets (21). Leng et al. illustrated a 24% reduction in methane production in Laos yellow cattle on biochar supplemented diets at 0.6% and a 40% when bound with potassium nitrate at 6% (20).

Biochar supplementation had varying effects on rumen fermentation parameters. Date palm seed-derived biochar had no significant effect on DMD (*p* = 0.522) or (*p* = 0.07) CFD degradability, but it significantly influenced fermentation parameters, including increased NH₃-N (*p* < 0.05) and TVFA (*p* < 0.01) levels, particularly at higher (*p* < 0.01) inclusion rates (4% level). Additionally, dietary biochar led to a significant linear decrease in ruminal pH, with the lowest values observed at the 4% level. Biochar can act as an electron shuttle in redox reactions within the rumen. This can influence the metabolic pathways of certain microbial populations, potentially diverting hydrogen away from methanogenesis (methane production) towards other pathways, such as propionate production (58). Propionate is a more energetically efficient volatile fatty acid (VFA) for the host animal. The highly porous nature of biochar provides extensive surface area for microbial colonization and adsorption (52). As previously mentioned, biochar contains lignin, a hydrophobic, amorphous polymer with a very high molecular weight. Lignin’s structure includes an aromatic substructure and various functional groups (59).

Some theories suggest biochar might directly capture or bind gases like methane and CO2 to some extent; however, this needs further confirmation. Recent findings indicate that dietary biochar in ruminant diets has shown variable effects, with some *in vivo* studies reporting no significant impact on animal performance, rumen fermentation, or methane emissions in lactating Holstein dairy cows (12). These inconsistencies are likely due to differences in biochar characteristics (such as dosage, source material, and composition), the type of basal diet, and the physiological status of the animals studied. However, in the current study, the inclusion of PSB-derived biochar in camels diet at 1, 2, and 4% levels significantly (*p* < 0.01) increased concentrations of short-chain fatty acids (SCFAs), metabolizable energy (ME), net energy for lactation (NEL), and organic matter degradability (OMD), while microbial crude protein (MCP, mg/g DM) and purine derivatives (PF, mg TDOM/mL gas) were not significantly affected by the addition of PSB to camel diets (*p* > 0.05). These improvements, particularly at the 1 and 2% inclusion levels, suggest that PSB in camels’ diet can enhance animal productivity. The observed increase in SCFAs during *in vitro* fermentation reflects a more efficient rumen fermentation process, as SCFAs are key energy substrates that support optimal growth, productive performance, and reproductive efficiency in ruminants (60). Moreover, the observed increase in total volatile fatty acids and short-chain fatty acids suggests enhanced fermentative activity, likely driven by improved microbial stabilization and nutrient utilization facilitated by biochar’s porous structure and surface chemistry (61).

The relationship between diet and SCFAs plays a crucial role in maintaining a healthy gut microbiota. SCFAs support microbial diversity and intestinal barrier integrity in healthy animals, while also enhancing gut resilience under acidic conditions (62). Supplementation of date palm seed-derived biochar in sheep diets has been shown to significantly improve growth rates, likely due to enhanced nutrient digestibility and improved rumen fermentation dynamics (13). These benefits may result from biochar’s ability to influence the passage of digesta through the gastrointestinal tract, thereby promoting more efficient digestion and potentially suppressing harmful bacterial populations (63). Bacteroidetes play a crucial role in methane generation by producing ample VFAs, thus aiding in anaerobic hydrolysis (64). Furthermore, the addition of biochar was observed to stimulate denitrification (the conversion of nitrate to dinitrogen) (65). This was evidenced by the presence of terminal electron acceptors and facilitate nitrate reduction.

Despite these promising findings, biochar supplementation did not significantly affect daily dry matter intake, milk yield, or feed conversion ratio (FCR) in sheep (19). Nonetheless, the current *in vitro* study provides valuable insights into the potential of date palm seed-derived biochar in camel diets as a sustainable feed additive for reducing methane emissions while enhancing nutrient utilization and animal performance. However, certain limitations must be acknowledged. Primarily, since the study focused only on camels, the generalizability of these results to other ruminant species may be limited. A limitation of this study was the need for further research on biochar inclusion in other animals to confirm its beneficial effects on the rumen ecosystem. Future *in vivo* studies involving multiple species are warranted to elucidate the underlying mechanisms of biochar’s effects and to establish optimal inclusion rates for effective methane mitigation, ultimately contributing to more climate-resilient livestock systems.

## 5 Conclusion

In the era of climate change, camels are gaining significant attention as a promising livestock species due to their remarkable adaptability to harsh environmental conditions. As camel farming expands, it’s crucial to find ways to sustain production while reducing greenhouse gas emissions from these animals in extensive systems. The results of this *in vitro* study found that PSB significantly increased total gas production and short-chain fatty acid concentrations, with the most notable improvements at 2% PSB inclusion. Importantly, methane emissions were markedly reduced, with the most substantial decrease (approximately 40%) observed at 1% PSB inclusion. Fermentation profiles were improved, as indicated by elevated volatile fatty acid levels and moderate shifts in rumen pH and ammonia concentrations. Digestibility and energy utilization metrics (ME and NEL) were also enhanced, without negative effects on microbial protein synthesis. These findings highlight PSB’s potential as an eco-friendly strategy to mitigate GHG emissions from camels while simultaneously boosting rumen fermentation and feed efficiency. Further research is needed to clarify the mode of action of biochar in *in vivo* experiments, specifically examining its effects on various physiological pathways and other reproductive and productive traits.

## Data Availability

The original contributions presented in the study are included in the article/supplementary material, further inquiries can be directed to the corresponding authors.

## Footnotes

^1^ https://linahfarms.com/?srsltid=AfmBOorVRJBNBIV8wWvFmNe-c4BVDIK9ZdW12kzNs5_uSg_GvxS_T2te
